# Membrane Permeabilization by *Bordetella* Adenylate Cyclase Toxin Involves Pores of Tunable Size

**DOI:** 10.3390/biom9050183

**Published:** 2019-05-10

**Authors:** David González-Bullón, Kepa B. Uribe, Eneko Largo, Garazi Guembelzu, Aritz B. García-Arribas, César Martín, Helena Ostolaza

**Affiliations:** Biofisika Institute, (UPV/EHU, CSIC) and Department of Biochemistry and Molecular Biology, University of Basque Country (UPV/EHU) Aptdo. 644, 48080 Bilbao, Spain; david_go88@hotmail.com (D.G.-B.); kepa.belloso@ehu.eus (K.B.U.); eneko.largo@ehu.eus (E.L.); garazi13@gmail.com (G.G.); aritzgarciaar@hotmail.com (A.B.G.-A.); cesar.martin@ehu.eus (C.M.)

**Keywords:** lipid-protein interactions, pore-forming proteins, protein toxins, membrane permeabilization, model membranes, atomic force microscopy

## Abstract

RTX (Repeats in ToXin) pore-forming toxins constitute an expanding family of exoproteins secreted by many *Gram*-negative bacteria and involved in infectious diseases caused by said pathogens. Despite the relevance in the host/pathogen interactions, the structure and characteristics of the lesions formed by these toxins remain enigmatic. Here, we capture the first direct nanoscale pictures of lytic pores formed by an RTX toxin, the Adenylate cyclase (ACT), secreted by the whooping cough bacterium *Bordetella pertussis*. We reveal that ACT associates into growing-size oligomers of variable stoichiometry and heterogeneous architecture (lines, arcs, and rings) that pierce the membrane, and that, depending on the incubation time and the toxin concentration, evolve into large enough “holes” so as to allow the flux of large molecular mass solutes, while vesicle integrity is preserved. We also resolve ACT assemblies of similar variable stoichiometry in the cell membrane of permeabilized target macrophages, proving that our model system recapitulates the process of ACT permeabilization in natural membranes. Based on our data we propose a non-concerted monomer insertion and sequential mechanism of toroidal pore formation by ACT. A size-tunable pore adds a new regulatory element to ACT-mediated cytotoxicity, with different pore sizes being putatively involved in different physiological scenarios or cell types.

## 1. Introduction

Adenylate cyclase toxin (ACT or CyaA) is crucial for colonization of the respiratory tract and disease establishment by *Bordetella pertussis*, the bacterium causative of whooping cough [1,2]. Cytotoxicity by ACT results from the synergy between toxin`s two main activities, production of supraphysiological cAMP levels by its adenylate cyclase domain (AC domain), and permeabilization, induced by its pore-forming domain (hemolysin domain), which debilitate the host defenses. 

ACT is member of an extensive family of toxins, referred to as RTX (Repeat in ToXin) [3,4]. Synthesis, maturation, and secretion of ACT are determined by the *CyaCABD* operon [5]. Gene product A is a 1706 amino acid polypeptide corresponding to a protoxin (pro-ACT) that matures in the bacterial cytosol to the active form by CyaC-directed acylation at two internal lysine residues (Lys 863 and Lys 913) [6]. ACT is then secreted across both membranes by the type I secretion system (CyaB, CyaD, and CyaE proteins). ACT is distinguished from the rest of RTX toxins by bearing a cell-invasive N-terminal enzymatic adenylate cyclase (AC) domain (∼364 residues) fused to a C-terminal RTX hemolysin moiety [5]. The catalytic AC domain converts ATP into cAMP [7]. The C-terminal RTX moiety (∼1342 carboxy-proximal residues) is responsible for the translocation of the AC domain across the host plasma membrane and for the hemolytic properties of ACT. This RTX moiety further consists of: a translocation region, spanning residues 365 to 500, which has been directly involved in the transport of the AC domain across the plasma membrane; a hydrophobic domain, spanning residues 500 to 700, containing several hydrophobic/amphipathic α-helical segments, which has been involved in pore formation by ACT [5,8]; an acylation region spanning residues 750 to 1000 that contains the two acylation sites (Lys, 863 and Lys 913) [6]; and a calcium-binding RTX domain, between residues 1008 and 1590, which harbors the characteristic Gly- and Asp-rich nonapeptide tandem repeats that form the numerous (∼40) calcium-binding sites of ACT—hallmark of ACT membership to the RTX family [9]. All ACT biological activities strictly depend on physiological (>0.3 mM) concentrations of free calcium ions in order to fold into an active toxin [10,11]. 

ACT targets primarily myeloid phagocytic cells that possess the CD11b/CD18 integrin, which acts as toxin receptor [12], although ACT can also efficiently intoxicate a variety of cells lacking the integrin, such as erythrocytes or epithelial cells, likely through a direct interaction with their plasma membrane [5]. To generate cAMP inside the target cell, ACT binds to the cell membrane and translocates directly its AC domain across the plasma membrane by a unique mechanism that involves an intrinsic ACT phospholipase A activity recently discovered in our laboratory [13,14]. Once in the cytosol, the AC domain catalyzes the unregulated conversion of intracellular ATP to cAMP in a reaction that is stimulated by eukaryotic calmodulin [5]. ACT can also form pores in membranes that account for the hemolytic activity of the toxin on erythrocytes [15,16]. 

ACT’s contribution to pore formation in membranes was concluded from haemolysis assays with osmotic protectants and conductance measurements in black lipid membranes [15,16,17]. ACT pores were described as transient small cation-selective channels, estimating a pore diameter of 0.6–0.8 nm, far from the pore sizes estimated for other homologous RTX toxins such as the *Escherichia coli* hemolysin that forms pores of 2.0–3.0 nm of diameter [15,16,17]. Haemolysis by ACT has been shown to be a highly cooperative event (Hill number ≥ 3) and slow kinetics, with a lag time of >1 h [18], what has led to assume that pore-forming activity of ACT involves toxin oligomerization. Recently, diverse ACT species with apparent molecular masses of 200, 300, 410, and 470 kDa, were detected by immunolabeling and blue-native polyacrylamide gel electrophoresis BN-PAGE of erythrocytes membranes treated with the toxin, attributing some of them (410 and 470 kDa) to oligomers of the 200 kDa ACT toxin [19]. 

Against the view that ACT and the other RTX toxins form discrete-size proteinaceous pores in membranes, additional studies, including our own data, have revealed that the RTX-induced perturbation in membranes depends on a number of factors, including membrane lipid composition, temperature, time, and toxin concentration [20,21]. This suggests that rather than being a static process, permeabilization by RTX toxins may be a complex, dynamic process involving membrane remodeling processes, accompanied by transient formation of non-lamellar structures in the membrane [21,22,23]. In the present study we have used a minimal model system along with fluorescence and atomic force microscopy techniques to investigate in detail the process of membrane permeabilization by ACT, and we capture what are arguably the first direct pictures of lytic pores formed by ACT in membranes. Our results support a new model for the ACT-mediated lesions in biological membranes that might be extensive to other toxins in the RTX family. 

## 2. Materials and Methods

### 2.1. Antibodies and Reagents

Anti-adenylate cyclase toxin RTX domain mouse monoclonal antibody (MAb 9D4) was from Santa Cruz Biotechnology (Santa Cruz, CA, USA); anti-mouse FITC, Hoechst, and Mitotracker were from Invitrogen, Molecular Probes (Carlsbad, CA, USA). 

### 2.2. ACT Purification

ACT was expressed in *Escherichia coli* XL-1 blue cells (Stratagene) transformed with pT7CACT1 plasmid, kindly provided by Dr. Peter Sebo (Institute of Microbiology of the ASCR, v.v.i., Prague, Czech Republic) and purified as described by Karst et al. [24]. 

### 2.3. Cell Culture

J774A.1 murine macrophages (ATTC, number TIB-67) were cultured at 37 °C in DMEM supplemented with 10% (*v*/*v*) heat-inactivated fetal bovine serum (FBS), 100 U/mL penicillin, 100 μg/mL streptomycin and 4 mM l-glutamine in a humidified atmosphere with 5% CO_2_. 

### 2.4. Permeabilization Measured as Influx of Fluorescent Solutes to the Interior of Giant Unilamellar Vesicles (GUVs)

Giant unilamellar vesicles (GUVs) of dioleylphosphatidylcholine (DOPC) were prepared using the electroformation method developed by Angelova and Dimitrov [25]. A special temperature-controlled chamber previously described by Bagatolli and Gratton [26] was used following the same protocol as in Montes et al. [27]. Briefly, 6 μg of lipid mixture dissolved in chloroform were spread on platinum electrodes in the electroformation chamber and allowed to dry, before immersion in 200 µL sucrose (300 mM in H_2_O). Electroformation proceeded for 2 h at 10 Hz, followed by 40 min at 2 Hz. Rhodamine-PE (0.25%) was used to label the GUVs. After vesicle formation the chamber was left to settle at room temperature. Then, for the visualization of the influx of different fluorescent solutes to the interior of the GUVs, the chamber was placed on an inverted confocal fluorescence microscope (Nikon Eclipse TE-2000, Nikon, Nikon Instruments, Tokyo, Japan). The excitation wavelengths were 488 nm (Alexa Fluor 488, Molecular Probes, ThermoFisher, Spain), 514 nm (Rhodamine-PE, Molecular Probes, ThermoFisher, Spain), and 490 nm (FITC-Dextrans, Molecular Probes, ThermoFisher, Spain). The fluorescence signal was collected into two different channels with band pass filters of 515/30 nm and, 590/94 nm. The objective used was a 63X oleo immersion with a NA of 1.2. Image treatment was performed with the Fiji software (https://imagej.net/) [28] The degree of GUV filling (P) was calculated as,

(1) $$P=\frac{F_{int}}{F_{out}}\times 100$$

where *F*_int_ and *F*_out_ are the average fluorescence intensities inside and outside a GUV at time *t*. We arbitrarily set to <20% the threshold for classifying GUVs as non-permeabilized. Several hundred GUVs were analyzed per experiment (≈200 per condition and experiment).

### 2.5. Atomic Force Microscopy (AFM): Sample Preparation, Measurements and Topographic Analysis

AFM measurements were carried out on ACT POPC (1-palmitoyl-2-oleyl-phosphatidylcholine) proteoliposomes extended on a thermostated mica support. For that we adapted the well-known vesicle adsorption method of McConnell et al. [29] to our experimental requirements. Firstly 125 µM of freshly prepared liposomes in Tris buffer (20 mM Tris-HCl, 150 mM NaCl, and pH = 8.0) supplemented with 5 mM CaCl_2_ were incubated with ACT at a protein:lipid ratio of 1:2000 during 10 min at 37 °C in vigorous agitation. Then formed proteoliposomes were centrifuged at 13,000× *g* for 15 min at 4 °C to discard aggregates and the supernatant was added on top of previously prepared 1.2 cm^2^ freshly cleaved mica substrate mounted onto a BioCell (JPK Instruments, Berlin, Germany) coverslip-based liquid cell for atomic force microscopy measurements. An extra 100 µL of Tris buffer (without calcium) was added. Vesicles were left to adsorb and extend onto the mica substrate increasing temperature by 4 °C per 5 min until a sample temperature of 37–42 °C was reached. Then, the sample was kept for 30 min at 37 °C. Later, another 30 min were left for the samples to equilibrate at room temperature, eliminating then the non-adsorbed vesicles by washing the samples 10 times with Tris buffer with 10 mM CaCl_2_. A small amount of buffer was always left on top of the structure in order to maintain the Supported Lipid Bilayers (SLB) hydrated at all time. The BioCell was set to 23 °C and the planar bilayers were left to equilibrate for 15 min prior to AFM measurements using the taping mode. Control experiments were carried out extending POPC liposomes without toxin onto the mica substrate. In this case, the temperature ramp was extended to 70 °C and maintained for additional 30 min prior to equilibration at 23 °C. 

The AFM measurements were performed on a NanoWizard II AFM (JPK Instruments, Berlin, Germany). MSNL-10 cantilevers (Brucker Billerica, MA, USA) with a spring constant of 0.03–0.5 N/m were used in either intermittent contact or contact mode scanning (constant vertical deflection) to measure the supported lipid bilayers SLBs. The tip radius was 2 nm (Nom) and 12 nm (Max). 512 × 512 pixel resolution images were collected at a scanning rate of 1–2 Hz (for intermittent contact mode) and 8–10 Hz (for contact mode) and line-fitted using the JPK Data Processing software as required.

### 2.6. Preparation of Large Unilamellar Vesicles (LUVs)

Large unilamellar vesicles (LUVs) were prepared by extrusion of multilamellar liposomes (MLV) using polycarbonate filters of 100 nm, following the method of Hope et al. [30]. 

### 2.7. Blue-Native (BN-PAGE) Electrophoresis and Western Blotting 

J774A.1 macrophages grown at confluency (≈1 × 10^5^ cells) were treated with ACT (20 nM) for different times at 37 °C, as indicated in the text. Then cells were washed three times with 5 mM EDTA in cold phosphate buffer (PBS) to eliminate unbound toxin. The cells were then scraped and centrifuged at 14,000× *g* for 10 min at 4 °C, and then pellets resuspended in solubilization buffer A (50 mM NaCl, 50 mM imidazole, 2 mM 6-aminohexanoic acid, 1 mM EDTA, and pH = 7.0) to which a mild detergent IGEPAL^®^ CA-630 (Sigma Aldrich, MO, USA) was added from a 20% stock in distilled water to a final concentration in the mixture of 5% (*w*/*v*). The resultant mixture was incubated for 5 min at room temperature to lyse the cells, and then centrifuged at 100,000× *g* for 15 min at 4 °C using the TLA-100 rotor (Beckman Coulter, CA, USA). The cellular debris was precipitated leaving in the supernatant cell membrane micelles, which was collected for its electrophoretic analysis. 

Large unilamellar vesicles (DOPC LUVs) were treated with ACT at different lipid:protein molar ratios (2500:1, 1000:1, and 250:1) for 30′ at 37°, as indicated in the text. Then samples were processed to eliminate unbound toxin following the procedure of flotation in sucrose gradient [31]. 

Native gradient gels were manually prepared in a Mini-PROTEAN 3 Multi-Casting Chamber coupled to a Model 485 Gradient Former, both provided by Bio-Rad (Irvine, CA, USA). The gels were prepared with an acrylamide gradient from 2 to 12% using two mixtures containing Gel buffer 3× (75 mM imidazole, 1.5 M 6-aminohexanoic acid, and pH = 7), AB3-mix (48 g acrylamide and 5 g bisacrylamide in 100 mL water) and glycerol at a final concentration of 20% (*v*/*v*) only in the case of the mixture with a higher percentage of acrylamide. To both mixtures 10% APS and TEMED were added to proceed with polymerization. 

Blue native polyacrylamide gel electrophoresis (BN-PAGE) was performed essentially as described by Wittig et al. [32]. NativePAGE ™ Sample Buffer (Thermo Fisher Scientific, MA, USA) was added to the samples and loaded onto the gradient gels, using as a cathode buffer a mixture of 50 mM tricine and 7.5 mM imidazole at pH = 7 and as anode buffer 25 mM imidazole at pH = 7. The gels were run at 4 °C to 150 V for 1 h followed by 250 V for another additional hour at 4 °C. Molecular size estimation was performed using calibration curves prepared with native protein standards (NativeMark ™ Unstained Protein Standard, Thermo Fisher Scientific, MA, USA). After electrophoresis, the separated proteins were electroblotted, using native cathode buffer, onto Immobilon-P membranes (Merck Millipore, Madrid, Spain)), and then detected by the indicated antibodies, and visualized by immunoperoxidase staining. The Quantity One^®^ Image Analyzer software program (Bio-Rad) was used for quantitative densitometric analysis.

### 2.8. Statistical Analysis

All measurements were performed at least 3 times, and results are presented as mean ± s.d. Levels of significance of the differences between groups were determined by a two-tailed Student’s *t*-test, and a confidence level of greater than 95% (*p* < 0.05) was used to establish statistical significance. When required (indicated in the Figure legends) data were analyzed using ANOVA followed by analysis by Tukeys test for pair comparison of subgroups; *, **, and *** represent *p*-value < 0.05, 0.01 and 0.001, respectively. The correlation analysis was assessed by the Pearson Product Moment correlation test [33]. 

## 3. Results 

### 3.1. Permeabilization of Giant Unilamellar Vesicles by ACT

To investigate in detail the mechanism of pore formation by ACT, we used the single giant unilamellar vesicle (GUV) methodology, in which the influx of fluorescent solutes of different sizes into the lumen of GUVs was directly monitored by confocal microscopy [34]. This is a reliable assay that has been previously used by other laboratories, and is particularly well suited to assess permeabilization features by lytic proteins and peptides [34,35,36,37]. As compared to bulk experiments of dye release (calcein, ANTS/DPX) in large unilamellar vesicles (LUVs), in which the information obtained is an average of the processes of the overall liposome population, the GUV methodology allows a more accurate estimation of the effective pore size (≈internal diameter) and on the permeabilization mechanism, because confocal microscopy allows the direct detection of individual vesicles [34,35,36]. 

**Figure 1:**
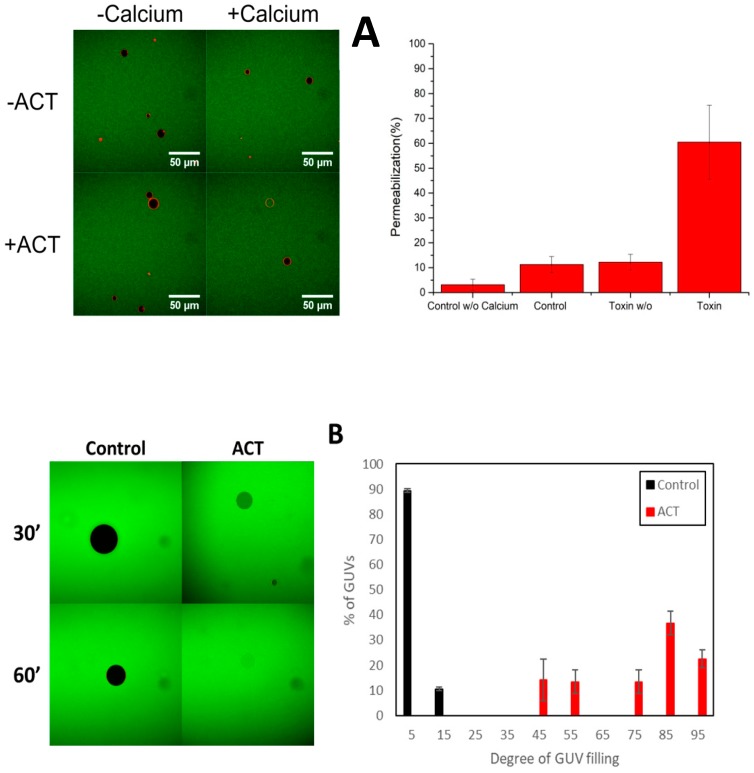
Calcium-dependent-permeabilization of dioleylphosphatidylcholine Giant Unilamellar Vesicles by ACT DOPC GUVs by ACT toxin. (**A**) Representative images of GUVs (black, if empty) in a solution of Alexa-Fluor488 (green) incubated in the absence or presence of 200 nM ACT and buffer without or with 10 mM CaCl_2_ (left panel). Images were taken 30 min after mixing the components. The internalization of Alexa-Fluor-488 to the lumen of GUVs (green) corresponds to permeabilized vesicles. GUV composition was DOPC. In the right panel the total percentage of permeabilization after 30′ for each condition (± ACT ± CaCl_2_) is depicted. In this case the threshold filling value to discriminate between permeabilized and non-permeabilized vesicles has been the 40%. (**B**) Distribution of the degree of filling of individual GUVs to Alexa-Fluor-488 (right panel), after treatment with 200 nM ACT for 30 min in buffer with 10 mM CaCl_2_. The degree of filling was calculated for each individual vesicle, from confocal images as the one shown in the left pane. In each of three experiments, a minimum of 250 vesicles were analyzed per condition. Error bars represent S.D.

Figure 1 shows images from a representative experiment in which we used GUVs of dioleylphosphatidylcholine, DOPC. The external fluorophore was Alexa-488 (stokes radius 5.8 Ȧ) [38]. The lack of fluorescence inside the vesicle (black color) indicates that it is impermeable to the external fluorescent molecule (green color). The pictures were taken after 30′ incubation, for control GUVs without toxin and ACT-treated vesicles in buffer with 10 mM calcium. The ACT concentration used was 200 nM, which is within the concentration range typically used to assess the ACT pore-forming activity in hemolysis assays with red blood cells [15,16,19,39] and similar to the concentrations used previously in experiments of solute efflux from large unilamellar liposomes (LUV) in our laboratory [20]. The images show that upon toxin treatment the GUVs became green-colored most likely due to membrane permeabilization by ACT pores that allow entry of Alexa-488 into the lumen of the GUVs, whereas control empty vesicles remained black colored. The same assay was also performed in buffer without calcium, in which it is expected that the calcium-dependent pore-forming activity of ACT will be inhibited [10,16]. As shown in Figure 1A, in absence of calcium the GUVs remained impermeable to the probe, thus corroborating that ACT permeabilizes the vesicles only in presence of the divalent cation, and all together proving that the observed GUV permeabilization reflects really the calcium-dependent pore-forming activity of ACT on membranes. 

Next we calculated the filling degree for each single vesicle measuring the average fluorescence intensity of Alexa-Fluor-488 inside the individual vesicles and in the external medium for GUVs treated with ACT (200 nM), and arbitrarily classified the GUVs with permeabilization values lower than 20% as non-permeabilized. For the statistical analysis, we sampled ≈200 vesicles in each experiment and repeated each experiment three times. The distribution of the degree of filling at single-GUV level provides information on the permeabilization mechanism (either “all-or-none” or “graded”) [34,35]. If the vesicles in the sample exhibit only two states, either impermeable or totally permeabilized, then the permeabilization mechanism would correspond to the “all-or-none”, but if the individual GUVs in the sample exhibit a varying degree of filling, then the permeabilization would follow the “graded” mechanism. All-or-none permeabilization is usually accompanied by the formation of stable discrete pores, and is characterized by strong cooperativity in permeabilization. By contrast, graded permeabilization is not cooperative, and exhibits slower kinetics, which leads to partial filling of the individual liposomes [34,35]. Figure 1B shows the distribution of filling degrees for the individual vesicles in the ensemble of GUVs, which strongly suggests that ACT follows a graded mechanism of permeabilization, since the vesicle population spans a large range of filling degrees. 

### 3.2. ACT Forms Growing-Size Pores, Which Depend on Toxin Concentration and Incubation Time 

The “graded” mechanism of permeabilization has been associated to the generation in the membrane of metastable structures such as toroidal or proteolipidic pores described for several amphipatic antimicrobial peptides [40]. This kind of structure is very sensitive to parameters such as protein concentration and incubation time. We tested whether any of these parameters contributed to ACT membrane-permeabilizing activity. 

**Figure 2:**
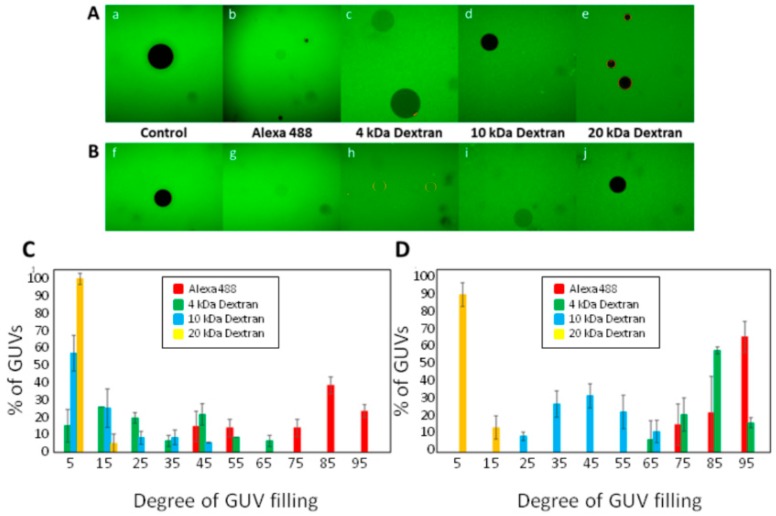
Permeabilization by ACT of GUVs to fluorescently-labeled solutes with diverse molecular sizes. Representative images of GUVs (gray-black) in solutions containing Alexa-Fluor-488, 4 kDa-FITC-Dextran, 10 kDa-FITC-Dextran or 20 kDa-FITC-Dextran incubated in the absence (control), or presence of 200 nM ACT. Images were taken 30 min (sub-panels a–e) (**A**) or 1h (sub-panels f–j) (**B**) after mixing the components. The filling of the lumen of GUVs with the corresponding fluorescent probe (green) corresponds to permeabilized vesicles. GUV composition was dioleylphosphatidylcholine (DOPC). Distribution of the degree of filling of individual GUVs to Alexa-Fluor-488, 4 kDa-FITC-Dextran, 10 kDa-FITC-Dextran or 20 kDa-FITC-Dextran, after treatment with ACT for 30 min (**C**) or for 1 h (**D**). In each of three experiments ≈200 vesicles were analyzed per condition.

To determine the possible effect of the incubation time in the size of the ACT pores we examined the permeability of the GUVs to solutes of different sizes (Alexa Fluor-488, 4 kDa FITC-Dextran, 10 kDa FITC-Dextran and 20 kDa FITC-Dextran) upon treatment with a single ACT dose (200 nM) for two different incubation times, 30′ and 60′, at RT. We took images by inverted fluorescence confocal microscope to check for vesicle integrity and vesicle filling (Figure 2). As shown in Figure 2A subpanels a–e none of the fluorescent solutes tested, except Alexa Fluor-488, was effectively internalized into the GUVs upon incubation with ACT in the short time (30 min). However, after 60′ all of them, except the 20 kDa FITC-Dextran, entered the GUVs (Figure 2B sub-panels f–j), though there were differences in the respective permeabilization extents, which directly correlated with the respective molecular masses of the probes. These data suggested that ACT formed in the GUVs small lesions that grow with time. From the hydrodynamic radii of the FITC-Dextrans of 4 and 20 kDa, ≈1.4 and ≈3.3 nm, respectively, it can be estimated the approximate effective diameter of the perturbation induced by ACT (200 nM) in the GUVs (upon 1h at RT) which would be between 2.8 nm and 6.6 nm. This indicates that ACT may form lesions larger than previously anticipated, that might even allow the passage of molecules such as small proteins (cytochrome c, diameter 3.1 nm), while vesicle integrity is preserved. From Figure 2C,D, which show the distribution of filling degrees for the individual vesicles after 30 and 60 min and for the different fluorescent solutes, we could infer, and corroborate, the conclusion we achieved for Alexa Fluor-488, this is, the graded mechanism followed by ACT in permeabilizing the GUVs.

**Figure 3:**
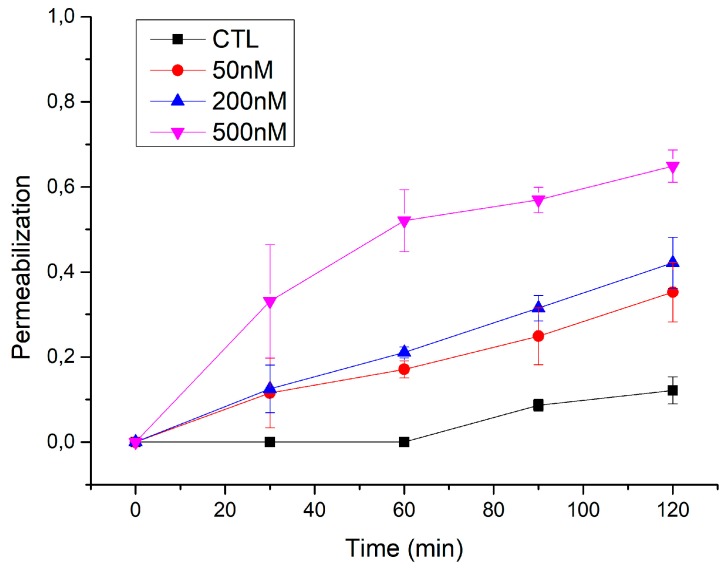
Permeabilization of GUVs by ACT as a function of the incubation time and the toxin concentration. Filling of DOPC GUVs with 10 kDa-FITC-Dextran was determined after incubation of the vesicles with three different ACT concentrations (50, 200, and 500 nM) and for different incubation times (30′, 60′, 90′, and 120′). The degree of filling was calculated as described in the Methods section.

To determine the possible effect of the toxin concentration on the size of the ACT pores we tried three different toxin concentrations (50, 200, and 500 nM) on the permeabilization of GUVs to a large solute, the 10 kDa-Dextran (≈4.6 nm of diameter), as a function of time (Figure 3 and Figure S1). As shown in the Figure 3, for the lower ACT concentrations tested (50 and 200 nM), the percentage of internalized Dextran-10 was almost null at the shortest incubation time (30 min), and increased slightly and almost linearly at longer incubation times. Only after ≈2 h this large Dextran entered into the ACT-treated GUVs in a considerable extent, of about ≈30–40%. However, at an ACT concentration of 500 nM, a similar degree of vesicle filling was achieved after only 30 min incubation with the toxin, and after 2 h the filling degree was of about 60% (Figure 3 and Figure S1). All together our findings indicated that ACT forms in membranes growing size pores that, depending on the toxin concentration and the incubation time may initially be small pores, permeable perhaps only to small solutes/ions, and evolve, at long incubation times or high toxin concentrations, into holes large enough so as to allow the flux of large molecular mass solutes (10 kDa Dextran), while vesicle integrity is preserved. This was a further indication that the ACT-induced openings in membranes, rather than being fixed-size pores, may be large and formed through a mechanism that may involve lipids.

### 3.3. Analysis by BN-PAGE and AFM of ACT Assemblies in Phosphatidylcholine Membranes

**Figure 4:**
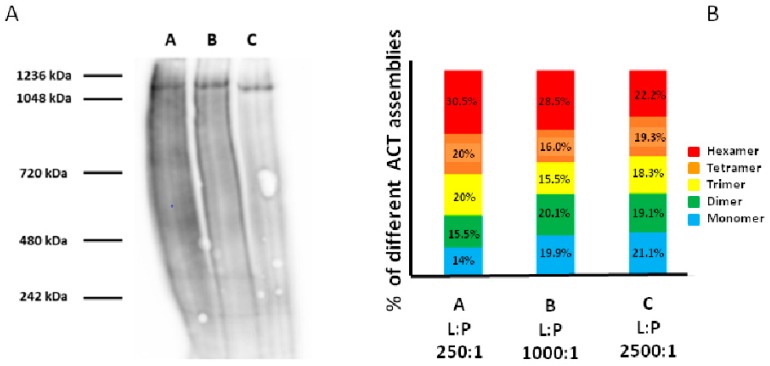
Analysis by BN-PAGE of ACT multimers in LUVs. Presence of possible multimeric ACT assemblies was detected by blue-native electrophoresis (BN-PAGE) of ACT-treated DOPC large unilamellar liposomes. (**A**) The lipid vesicles were incubated with the toxin for 30 min at 37 °C at three lipid: protein ratios (mol ratio) 250:1 (track A), 1000:1 (track B), and 2500:1 (track C), then samples were processed to remove the unbound toxin, and then run in a blue native gel (3–12% acrylamide gradient) and the corresponding blotted membrane stained with an anti-ACT MAb (9D4). (**B**) Quantification by densitometry of the relative intensities of the ACT protein bands detected in the blotted membrane with the 9D4 monoclonal antibody.

To detect possible multimeric ACT assemblies, blue-native electrophoresis (BN-PAGE) of ACT-treated liposomes was performed (Figure 4). For reasons of handability we used large unilamellar vesicles (DOPC LUVs). The lipid vesicles were incubated with the toxin for 30 min at 37 °C at three different toxin concentrations 250:1, 1000:1, and 2500:1, expressed as lipid:protein molar ratios, then the samples were processed to remove the unbound toxin, and then run in a blue native gel (3–12% acrylamide gradient) and the corresponding blotted membrane stained with an anti-ACT MAb (9D4) (Figure 4**,** left panel). Several spots, corresponding, the most clear of them, to different apparent molecular masses, ranging from ≈550 to ≈1200 kDa, could be visualized in the membrane, but other smaller more diffuse spots were also detected (Figure 4, left panel), corroborating our hypothesis that ACT might form multimeric assemblies, with variable stoichiometry. Densitometry of the main bands detected in samples corresponding to a lipid:protein mole ratio of 1000:1 (track B, left panel of Figure 4) gave an approximate relative abundance of ≈20% of a ≈200 kDa band (monomer?), ≈20% of a ≈400 kDa band (dimer?), ≈15% of a ≈550 kDa band (trimer?), ≈16% of a ≈800 kDa band (tetramer?), and ≈28% of a ≈1200 kDa band (hexamer?) (Figure 4, right panel). Interestingly, incubation of the lipid vesicles with a higher or lower ACT concentration (lipid: protein 250:1 and 2500:1 lipid:protein molar ratio), shifted the assembly equilibrium to a higher or lesser proportions of the oligomeric forms, respectively (tracks A and C of Figure 4, respectively). These results reaffirmed our previous observation on the concentration dependence of ACT multimerization (Figure 3). 

**Figure 5:**
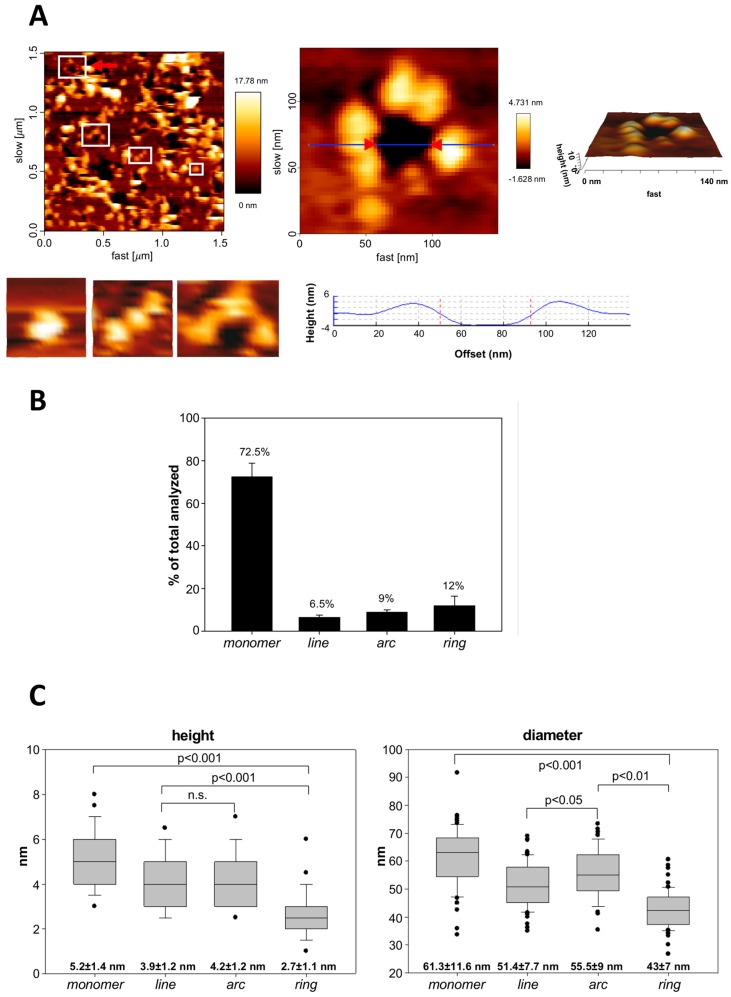
Analysis by atomic force microscopy of the assemblies formed by ACT in supported lipid membranes. (**A**) AFM image (left upper panel) of a supported lipid bilayer (SLB) prepared from ACT-containing proteoliposomes (POPC liposomes reconstituted with ACT). The red arrowhead points to a membrane pore that has been selected for a more detailed topographic analysis; in the image there are more pores, heterogeneous in size and shape; the edges of the pores present protrusions corresponding to ACT clusters. Below this image, other ACT assemblies are shown, such as a monomer, a line and an arc, which have been selected from other AFM images. On the right-hand part of panel A, a 3D AFM topography of the pointed ACT ring is shown in a greater detail. The image reveals a circular dark hole that spans the lipid membrane (below). ACT molecules around the pore rim (yellow-white) protrude 3.97 ± 1.02 nm above the membrane plane, as confirmed by the height profile shown below the image (corresponding to the blue line in the middle of the 2D image. (**B**) Quantitative analysis of the structures found for ACT on SLBs. Data show the percentage of each type of structure (monomer, line, arc or ring) in all the measurements. (**C**) Determination of the diameter and height (in nm) of the monomeric particle in each of the different ACT assemblies (monomer, line, arc or closed ring). Mean values are depicted as box-and-whisker plots (the ends of the whiskers represent standard deviations). A total of 973 particles were measured for these determinations.

To investigate in more detail the nanoscale organization of ACT assemblies at the membranes and whether such arrangements were related to the formation of functional ACT pores, we performed experiments of atomic force microscopy (AFM). In addition to a high spatial resolution, this technique provides also information about the 3D topography of the membrane surface. To this aim we prepared supported lipid bilayers from proteoliposomes containing ACT (2000:1 lipid:protein molar ratio) and we analyzed them with AFM (room temperature). Due to technical constraints of the AFM and to improve the stability of the liposomes and proteoliposomes extended onto the mica surface we used palmitoyloleylphosphatidylcholine (POPC) liposomes instead of DOPC vesicles to prepare ACT-containing proteoliposomes. Binding and permeabilization characteristics of ACT were very similar in POPC and DOPC liposomes. In contrast to control samples which exhibited largely flat membranes with few defects (Figure S2), the supported lipid bilayers containing ACT were rich in structures protruding several nanometers, from ≈3.0 to ≈8.0, from the membrane plane (Figure 5A). Consistent with the GUVs results and the BN-PAGE data, the detected ACT structures were heterogeneous in size, and very interestingly, they included lines, rings and arc-shaped arrangements of protein, in addition to abundant single dots (likely monomers), and some random aggregates (Figure 5A). Upon quantification of 973 particles, the relative percentage of each structure was determined: values of 72.5 ± 6.3% for monomers, 6.54 ± 1.0% for lines, 8.96 ± 1.0% for arcs and 12.02 ± 4.4% for closed rings being obtained (Figure 5B). Differences between the relative abundances as determined in the LUVs (BN-PAGE) or in the proteolipidic preparations (AFM) were detected, observing a greater proportion of higher order structures in LUVs (≥80%) relative to the proteolipidic preparations (≈28%), which, besides differences in sample preparation, might be reflecting temperature-dependent differences in the oligomerization kinetics of ACT, since the samples for the BN-PAGE measurements were incubated at 37 °C, while the AFM images were taken at ≈23 °C.

Other parameters determined from the AFM images were the height and the diameter of the monomeric particle in each type of ACT assembly (monomer, line, arc and ring) (Figure 5C). The data were very revealing, and indicated that, whereas the single ACT monomers had a broad and heterogeneous distribution of heights, between ≈3 nm and ≈8 nm (over the plane of the membrane), the closed rings had a much narrower and homogeneous distribution, predominating heights of around 2.0–3.0 nm. The lines and arcs appeared to have an intermediate height distribution (≈3.0–6.0 nm), and in this case the more frequent heights were of about ≈4 nm. These height differences might reflect different penetration degrees of the ACT structures into the lipid bilayer, and suggest that to assemble into higher order structures (oligomers), the ACT monomers might require a deeper insertion into the lipid bilayer. Regarding the diameter of the monomeric particle in the different ACT structures, a progressive decrease in particle size was observed, from ≈60 nm for the monomer alone, to ≈40 nm in the closed rings, passing through ≈50 nm in lines and arcs (Figure 5C and Figure S3). 

Assuming a diameter of ≈14–15 nm for the ACT monomer in solution, as recently determined by Small Angle X-Ray Scattering SAXS [41] our data suggested that the toxin would transit from a more or less “compact” state in solution, to a more “open” or “extended” conformation upon binding to the membrane. Note that in spite of the great variability, a direct correlation between the diameter of the ACT monomeric particle and the particle height can be seen (Figure S3) more clearly for the ACT free monomers and lines, so that, the larger the particle diameter, the greater the height, or vice versa, the smaller the particle diameter, the smaller the height. This would, in turn, indicate that the monomeric particle size (diameter) and its degree of penetration into the lipid bilayer are inversely correlated, so, the smaller the monomeric particle size, the greater its penetration degree. This effect might respond to different protein-lipid or protein-protein interactions, or reorganizations that the ACT polypeptide would undergo during the insertion process. The large variability of heights and diameters detected among the ACT monomers themselves is also interesting, which might reflect that ACT penetration into the lipid bilayer is a slow process, in which the different-height monomers might correspond to different insertion-intermediates. It might thus be anticipated that penetration into the lipid bilayer might be a rate-limiting step for pore formation by ACT. 

**Figure 6:**
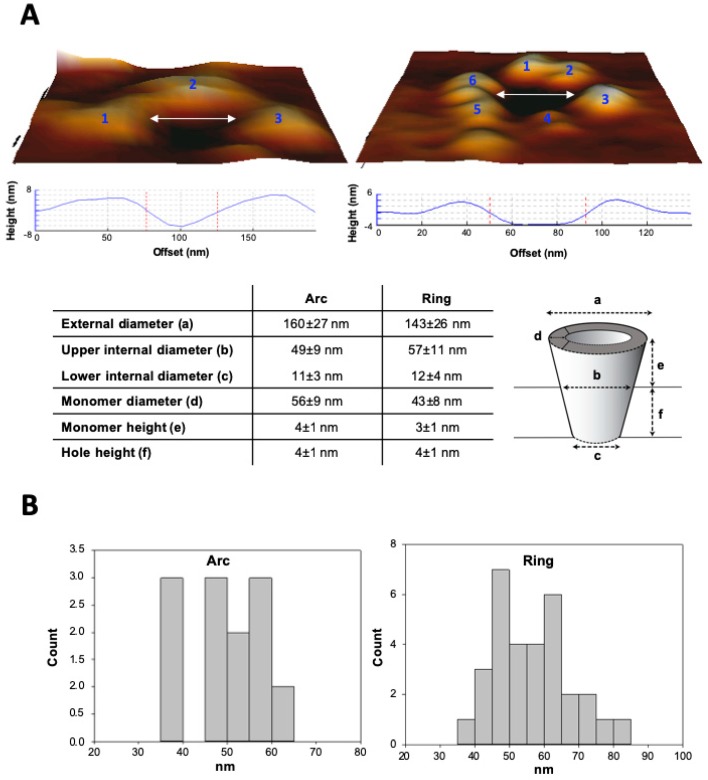
Three-dimensional atomic force microscopyAFM topography of ACT assemblies that pierce the lipid bilayer. (**A**) Detailed 3D topographic analysis of two types of ACT assemblies, arcs (left-hand side) and full rings (right-hand side), for which each constituent monomer has been numbered. Below, different parameters (external arc diameter, diameter, internal ring diameter, monomer diameter, monomer height and ring “hole” height) and their respective values are listed. (**B**) Measurement of the number of monomeric particles with a given diameter (in nm) in different ACT arc and ring assemblies.

Interestingly, full rings visualized in the AFM images showed unanticipated large apparent diameters that ranged from ≈40 nm to ≈80 nm in the more external side (upper vestibule) of the pore, and ≈20 nm in the deeper side of the hole (Figure 6). The idea that the ACT pore structures visualized here by AFM were indeed “real holes” was further verified and corroborated by determining the depth of the lesions, which ranged from ≈−3.0 to ≈−5.0 nm (with respect to the flat surface of the lipid bilayer) in both arcs and full rings. This hole depth fits well with membrane thickness, which is about 5.0 as determined in the control POPC bilayers (see Figure S2) and that is also in full agreement with the thickness value reported by others for PC bilayers [42,43]. This demonstrated that such ACT pore structures traversed the lipid bilayer, consistently with the membrane permeabilization determined in the model vesicles (Figure 1, Figure 2 and Figure 3). 

### 3.4. Permeabilization of Target Macrophages by ACT Directly Correlates with the Toxin Assembly into Oligomers of Variable Stoichiometry

**Figure 7:**
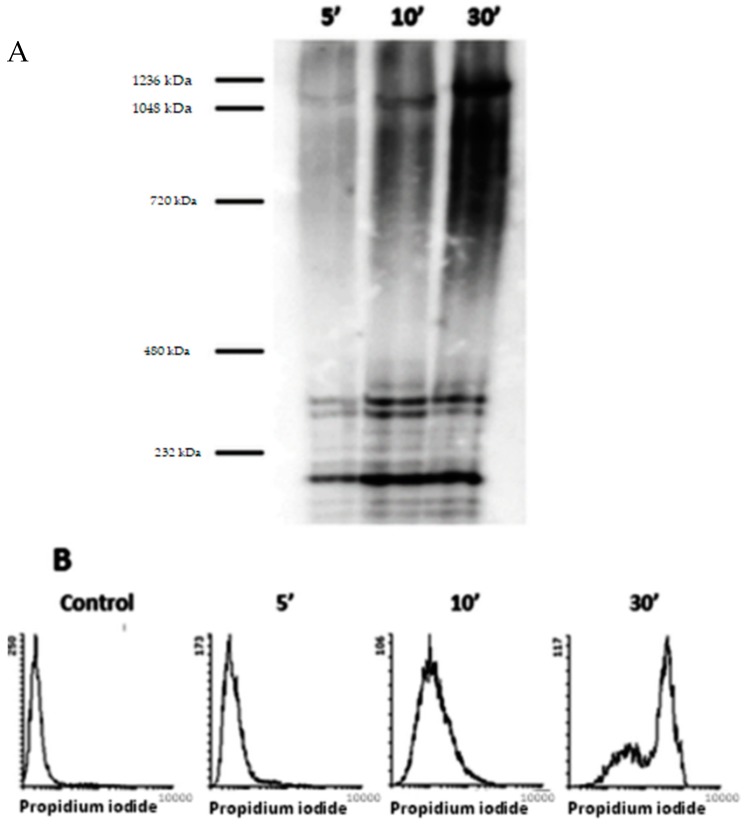
Analysis by blue native polyacrylamide gel electrophoresis BN-PAGE of target macrophages exposed to ACT for different incubation times and PI uptake as determined by flow cytometry. (**A**) J774A.1 cells (1 × 10^6^ cells/mL) were incubated with ACT (30 nM) at 37 °C for several incubation times (5–30 min) and then the separated membranes were electrophoresed by BN-PAGE, blotted into a polyvinylidene difluoride (PVDF) membrane and stained with anti-ACT MAb 9D4. Several protein bands of apparent high molecular masses were resolved, corresponding most likely to ACT oligomers of variable stoichiometry. (**B**) PI uptake into J774A.1 macrophages (1 × 10^6^ cells/mL) treated with ACT (30 nM) at 37 °C for several incubation times (5–30 min) as determined by flow cytometry.

To check whether ACT oligomerization is also involved in permeabilization of target cells we incubated CD11b/CD18-expressing J774A.1 cells (1 × 10^6^ cells/mL) with ACT (30 nM) at 37 °C, for different incubation times (5–30 min), and after extensive washing to eliminate unbound toxin, the cell membranes were electrophoresed by BN-PAGE and immunoblotted. The toxin concentration used was in the same range to the used previously by other authors to assess permeabilization effects of ACT on macrophages [15,39,44]. As shown in Figure 7, at the shortest incubation time (5 min) the main protein bands visualized in the nitrocellulose membrane had apparent molecular masses between ≈200 kDa and ≈400 kDa, corresponding likely to monomers and perhaps dimers of ACT. After longer incubation times (10 and 30 min) however, other bands with higher apparent molecular masses (≈800 kDa, ≈1000 kDa, and ≈1200 kDa) were detected (Figure 7A), suggesting a time-dependent ACT oligomerization. Importantly, the progressive appearance of toxin bands with higher apparent molecular masses showed a nice correlation with the progressive uptake of propidium iodide (PI) detected by flow cytometry in macrophages exposed to the same ACT concentration (30 nM) (Figure 7B), suggesting that cell permeabilization by ACT involves toxin assembly into oligomers of variable stoichiometry, thus corroborating the conclusions drawn from our model systems. The stoichiometry of the ACT oligomers in the macrophage membranes agreed well with the toxin oligomers visualized by AFM in the supported lipid bilayers (Figure 6) and by BN-PAGE of ACT-containing liposomes (Figure 4), and are also fully consistent with previous reports showing that hemolysis of red blood cells by ACT is a highly cooperative process with a cubic or higher power dependence with respect to toxin concentration [15,18]. Furthermore, we detected in the cell membrane of CR3(-) cells ACT oligomers of apparent molecular masses of ≈ 800 and 1000 kDa (Figure S4), very similar to the toxin multimers detected in the CR3-expressing J774A.1 macrophages. CR3(−) cells do not express the ACT receptor CR3 (CD11b/CD18) integrin, but are, at higher toxin concentrations, susceptible to permeabilization by ACT. From these data we discarded that ACT oligomerization was receptor-dependent, and concluded that similar ACT oligomers may form in membranes of both receptor-expressing and non-expressing cells.

## 4. Discussion

Lack of detailed molecular knowledge on the structure and characteristics of the lesions formed by RTX pore-forming toxins is a long-standing gap in the field. In this study, we present an exhaustive analysis of pores formed by ACT, using the single giant unilamellar vesicle (GUV) methodology and spectroscopic and atomic force microscopy techniques. This approach has allowed us to directly visualize the passage of differently sized fluorescent solutes across ACT pores, as well as to explore how different parameters such as toxin concentration and incubation time affect ACT pore size, and we have been able to visualize ACT molecules in membrane pore structures, showing that ACT forms heterogeneous assemblies of growing size that perforate the membrane. Our data may constitute the first direct proof of oligomeric lytic pores formed by a toxin from the RTX family in lipid bilayers, a task that had remained elusive for a long time, and suggest that ACT belongs to the very small set of proteins that can form variable-sized pores.

Since the early nineties the prevalent molecular model of cell permeabilization (haemolysis) by ACT has involved binding and insertion of an ACT conformer, apparently representing a pore precursor, that would oligomerize within the membrane of red blood cells forming discrete, protein-lined lytic pores that would apparently be of very small size (0.6–0.8 nm diameter) [16,17]. In contrast we show here that ACT may form, in cell-sized model systems of membranes (GUVs), an initial small lesion that increases in apparent diameter over time, becoming large enough so as to allow the influx of large molecular mass solutes (i.e., fluorescently labeled high molecular mass dextrans), while vesicle integrity is preserved (Figure 1, Figure 2 and Figure 3). Moreover, we show that the size of the ACT pore can be also regulated by toxin concentration (Figure 3). Similar conclusions had been noted by the group of Welch for the pores formed by the *Escherichia coli* HlyA hemolysin, a homologous toxin belonging also to the RTX family of toxins [21]. As far as we know, there is no precedent for a purely proteinaceous channel that undergoes changes in size, in a concentration-dependent manner, as described here for ACT. In contrast, toroidal or proteolipidic pores described for several amphipathic antimicrobial peptides, or pore-forming proteins such as pro-apoptotic Bax can be regulated by the lipid:protein molar ratio [34,40,45]. Our results thus indicate that ACT-induced openings in membranes, rather than being static fixed-sized small pores, may be large, heterogeneous and formed through a lipidic mechanism. Consistently, we determined here that ACT follows a “graded” mechanism of membrane permeabilization (Figure 1 and Figure 2). In agreement with our data, Fiser and Konopasek [46] previously determined a graded mechanism of permeabilization for ACT by using the method of bulk content release in LUVs (“requenching” method). 

Very interestingly, we provide AFM images showing ACT assemblies of heterogeneous architectures, in the forms of lines, arcs or closed rings, inserted in pure lipid vesicles made of phosphatidylcholine (Figure 5 and Figure 6), and we demonstrate that ACT arcs and rings pierce the membrane, corresponding thus to ACT lytic pores (Figure 6). These ACT arrangements exhibit unexpectedly wide and variable diameters, some of which show diameters of up to ≈20 nm in the narrowest part of the vestibule and ≈50–60 nm in the more external part limiting the aqueous medium (Figure 6). These AFM data are thus consistent with the size data obtained with GUVs, which show an efficient filling of the vesicles with fluorescent dextrans of large molecular masses such as the 10 kDa FITC-Dextran (stokes radius 2.36 nm). We can thus affirm that depending on the toxin concentration ACT may form large membrane pores of several nanometers wide, greater than previously reported [47]. Such large pores had indeed been reported only for cholesterol-dependent cytolysins such as listeriolysin, streptolysin or pneumolysin, which create large holes of β-structure [37,48,49]. 

Besides these larger toxin assemblies, we have also detected by AFM abundant free ACT monomers inserted into the lipid membrane. Both the ACT assemblies (lines, arcs and rings) as well as the monomers detected in our pictures present great height variability in their constituent monomeric particles, with height values from ≈3.0–6.0 nm for the monomeric particles in lines and arcs, to values of ≈2.0–3.0 nm for the closed rings. Interestingly, the highest height variability is shown by the free ACT monomers, which protrude between ≈3.0 to 8.0 nm from the plane of the lipid bilayer (Figure 6). Likely, these differences in the monomer height reflect different penetration degrees of the ACT monomeric particles into the lipid bilayer, and it appears that a correlation exist between the monomer insertion degree and the assembly order (ring > arc > line > monomer), suggesting that the deeper the ACT “monomeric particle” inserts, the larger is the oligomer that can be formed. All these data lead us to hypothesize that pore formation by ACT might follow the so-called “non-concerted pathway” [50,51] in which the water-soluble monomers bind first to the membrane in a likely fast step, and then membrane insertion of the monomer may take place earlier than (or concomitantly with) oligomerization, being possible to detect intermediate pore stages with lower stoichiometries. A non-concerted insertion model was previously proposed for pores formed by the propaptotic Bax protein, or by toxins of the actinoporin family that insert α-helices into the membrane [51]. This model of pore formation is associated with pores whose walls are lined both by lipids and proteins and it is characteristic of heterogeneous and flexible architectures [51]. The AFM data are thus fully consistent with the permeabilization data in GUVs, and reinforce the idea that ACT forms in membranes proteolipidic toroidal lytic pores. 

Canella et al. [41] have recently provided the first in solution structural characterization of the calcium-loaded monomeric ACT, which shows that the full length toxin adopts a compact and stable state with a mean diameter of ≈14–15 nm, in which the toxin’s calcium-binding C-terminal domain stabilizes the N-terminal catalytic domain [41]. Strikingly, the (total) diameter of the ACT monomer on the membrane as determined in our study, ranges from the ≈60 nm (free monomer), to the ≈50 nm (monomer in the lines and arcs), and to ≈40 nm (closed rings) (Figure 5C), suggesting that upon binding to the membrane, the ACT polypeptide would undergo a transition from a compact state in solution, to an open, extended state on the lipid surface. The initial binding and opening of the ACT monomer would be followed by protein–lipid interactions and subsequent reorganization of the ACT polypeptide leading to a progressively more inserted state (height of the monomeric particle in monomers goes from ≈8.0 nm to ≈3.0 nm, from the plane of the membrane) and this progressive insertion of the monomer chain, which is parallel to a decrease in particle diameter (Figure 5C and Figure S3), might bring the particles closer to each other, thus favoring protein–protein interactions and promoting ACT oligomerization (height values of the monomeric particle in the closed rings are ≈2.0–3.0 nm) and formation of larger lytic pores. The observed ACT lines and arcs (height values between ≈3.0–6.0 nm) might perhaps be, in this context, “intermediates” of oligomerization, suggesting a “sequential” mode of oligomerization [51], which involves the addition of units with the same stoichiometry (likely ACT monomers). This model has been described for proteins forming toroidal proteolipidic pores in membranes [51]. From our permeabilization and AFM data we hypothesize that the kinetics of ACT oligomerization may be directly conditioned by the kinetics of penetration into the lipid bilayer of the toxin monomers, being perhaps this step the rate limiting one for the assembly of oligomeric ACT lytic pores. 

Finally we show that in the cell membranes of target macrophages treated with ACT under permeabilization conditions, toxin assemblies with variable stoichiometry (Figure 7) are formed, very similar to the ones detected in model membranes and supported lipid bilayers. Moreover, we detect ACT oligomers of similar apparent molecular masses also in the cell membrane of CR3(−) cells (Supplementary Figure S4), which are devoid of the ACT receptor (CR3 or CD11b/CD18 integrin), suggesting that ACT oligomerization is receptor-independent, and that ACT may form similar ACT oligomers in the cell membranes of both receptor-expressing and non-expressing cells. The time-dependent appearance of ACT assemblies of apparent higher molecular masses nicely correlates with a progressive increase in the PI uptake by the cells (Figure 7), strongly suggesting that cell permeabilization by ACT is indeed carried out by oligomeric pore structures, and demonstrating that our results from model membranes recapitulate the pore formation by ACT in natural membranes. 

ACT oligomers detected here both in vitro (liposomes) and in cell membranes (J774A.1 cells and CR3(−) cells) have estimated molecular masses of ≈550, 800, 1,000, and 1,200 kDa, which may correspond to ACT trimers, tetramers, pentamers, and hexamers. The data are thus in full agreement with previous studies showing that haemolysis by ACT is a highly cooperative event with Hill number ≥ 3 [16]. Previously other group solved by BN-PAGE smaller ACT oligomers of apparent molecular masses of ≈410 and 470 kDa in toxin-treated erythrocytes, corresponding perhaps to ACT dimers [19]. In said experiments sheep erythrocytes were treated with ACT for 30′ and since for detecting hemolysis incubations of ≈180–240 min are usually used, it can be speculated that such smaller assemblies might become into larger ACT oligomers, similar to the ones detected in our present study, at longer toxin treatments. There is other early study on haemolysis by ACT [15] in which the authors conclude that the ACT pore size has to be ≤0.62 nm, since they observe that small sugars such as arabinose (75 mM) (estimated diameter ≈0.62 nm) protect red blood cells from hemolysis [15]. Here instead we have shown that ACT may form in membranes large holes of variable effective diameters of several nm (Figure 1, Figure 2 and Figure 3 and Figure S2). The question is whether these results are or not mutually excluding. We think that the growing size model we propose here for ACT pores may explain and reconcile these seemingly contrasting results, and so, it can be expected that large ACT pores would form whenever enough toxin concentrations and incubation times are used so as to allow toxin oligomerization (Figure 1, Figure 2 and Figure 3 and Figure S2). In the mentioned osmotic protection study [15], RBC were supposedly incubated with ACT for a very short time (15′) and then washed to continue with incubation for 5h in presence of the protectants [15]. A short exposition time and a washing step might have left less toxin molecules at the cell membrane, hindering large oligomers to be formed, and so only small (monomeric?) pores might have formed, that would be protectable by small sugars. 

In conclusion, we provide here for first time direct picture of lytic pore structures formed by an RTX toxin in biological membranes and mechanistic details on the process of pore formation; our data indicate that cell permeabilization and lysis by ACT is driven by toxin oligomeric pores of toroidal characteristics, which might likely have different architectures (arcs, closed rings) and variable stoichiometry, and that depending on several conditions (toxin concentration, cell type, etc.) they might become large enough so as to allow the efflux of solutes larger than mere ions. A tunable-sized pore adds a new regulatory element in ACT-mediated cytotoxicity, so that different pore sizes could be associated to different physiological scenarios or cell types.

## Supplementary Materials

The supplementary materials are available online at https://www.mdpi.com/2218-273X/9/5/183/s1.

## References

[1] Carbonetti N.H. (2010). Pertussis toxin and adenylate cyclase toxin: Key virulence factors of *Bordetella pertussis* and cell biology tools. Future Microbiol..

[2] Mattoo S., Cherry J.D. (2005). Molecular pathogenesis, epidemiology, and clinical manifestations of respiratory infections due to *Bordetella pertussis* and other Bordetella subspecies. Clin. Microbiol. Rev..

[3] Welch R.A. (1991). Pore-forming cytolysins of gram-negative bacteria. Mol. Microbiol..

[4] Welch R.A. (2000). RTX toxin structure and function: A story of numerous anomalies and few analogies in toxin biology. Curr. Top Microbiol. Immunol..

[5] Ladant D., Ullmann A. (1999). *Bordetella pertussis* adenylate cyclase: A toxin with multiple talents. Trends Microbiol..

[6] Hackett M., Guo L., Shabanowitz J., Hunt D.F., Hewlett E.L. (1994). Internal lysine palmitoylation in adenylate cyclase toxin from *Bordetella pertussis*. Science.

[7] Berkowitz S.A., Goldhammer A.R., Hewlett E.L., Wolff J. (1980). Activation of prokaryotic adenylate cyclase by calmodulin. Ann. N Y Acad. Sci..

[8] Vojtova J., Kamanova J., Sebo P. (2006). Bordetella adenylate cyclase toxin: A swift saboteur of host defense. Curr. Opin. Microbiol..

[9] Rose T., Sebo P., Bellalou J., Ladant D. (1995). Interaction of calcium with *Bordetella pertussis* adenylate cyclase toxin. Characterization of multiple calcium-binding sites and calcium-induced conformational changes. J. Biol. Chem..

[10] Hewlett E.L., Gray L., Allietta M., Ehrmann I., Gordon V.M., Gray M.C. (1991). Adenylate cyclase toxin from *Bordetella pertussis*. Conformational change associated with toxin activity. J. Biol. Chem..

[11] Hanski E., Farfel Z. (1985). *Bordetella pertussis* invasive adenylate cyclase. Partial resolution and properties of its cellular penetration. J. Biol. Chem..

[12] Guermonprez P., Khelef N., Blouin E., Rieu P., Ricciardi-Castagnoli P., Guiso N., Ladant D., Leclerc C. (2001). The adenylate cyclase toxin of *Bordetella pertussis* binds to target cells via the alpha(M)beta(2) integrin (CD11b/CD18). J. Exp. Med..

[13] González-Bullón D., Uribe K.B., Martín C., Ostolaza H. (2017). Phospholipase A activity of adenylate cyclase toxin mediates translocation of its adenylate cyclase domain. Proc. Natl. Acad. Sci. USA.

[14] Ostolaza H., Martín C., González-Bullón D., Uribe K.B., Etxaniz A. (2017). Understanding the mechanism of translocation of adenylate cyclase toxin across biological membranes. Toxins.

[15] Ehrmann I.E., Gray M.C., Gordon V.M., Gray L.S., Hewlett E.L. (1991). Hemolytic activity of adenylate cyclase toxin from *Bordetella pertussis*. FEBS Lett..

[16] Szabo G., Gray M.C., Hewlett E.L. (1994). Adenylate cyclase toxin from *Bordetella pertussis* produces ion conductance across artificial lipid bilayers in a calcium- and polarity-dependent manner. J. Biol. Chem..

[17] Benz R., Maier E., Ladant D., Ullmann A., Sebo P. (1994). Adenylate cyclase toxin (CyaA) of *Bordetella pertussis*. Evidence for the formation of small ion-permeable channels and comparison with HlyA of *Escherichia coli*. J. Biol. Chem..

[18] Gray M., Szabo G., Otero A.S., Gray L., Hewlett E. (1998). Distinct mechanisms for K+ efflux, intoxication, and hemolysis by *Bordetella pertussis* AC toxin. J. Biol. Chem..

[19] Vojtova-Vodolanova J., Basler M., Osicka R., Knapp O., Maier E., Cerny J., Benada O., Benz R., Sebo P. (2009). Oligomerization is involved in pore formation by Bordetella adenylate cyclase toxin. FASEB J..

[20] Martín C., Requero M., Masin J., Konopasek I., Goñi F.M., Sebo P., Ostolaza H. (2004). Membrane restructuring by *Bordetella pertussis* adenylate cyclase toxin, a member of the RTX toxin family. J. Bacteriol..

[21] Moayeri M., Welch R.A. (1994). Effects of temperature, time, and toxin concentration on lesion formation by the *Escherichia coli* hemolysin. Infect. Immun..

[22] Brown A.C., Boesze-Battaglia K., Du Y., Stefano F.P., Kieba I.R., Epand R.F., Kakalis L., Yeagle P.L., Epand R.M., Lally E.T. (2012). Aggregatibacter actinomycetemcomitans leukotoxin cytotoxicity occurs through bilayer destabilization. Cell Microbiol..

[23] Bakás L., Chanturiya A., Herlax V., Zimmerberg J. (2006). Paradoxical lipid dependence of pores formed by the *Escherichia coli* a-hemolysin in planar phospholipid bilayer membranes. Biophys. J..

[24] Karst J.C., Ntsogo Enguene V.Y., Cannella S.E., Subrini O., Hessel A., Debard S., Ladant D., Chenal A. (2014). Calcium, acylation, and molecular confinement favor folding of *Bordetella pertussis* adenylate cyclase CyaA toxin into a monomeric and cytotoxic form. J. Biol. Chem..

[25] Angelova M.I., Dimitrov D.S. (1986). Liposome electroformation. Faraday Discuss Chem. Soc..

[26] Bagatolli L.A., Gratton E. (1999). Two-photon fluorescence microscopy observation of shape changes at the phase transition in phospholipid giant unilamellar vesicles. Biophys. J..

[27] Montes L., Alonso A., Goñi F.M., Bagatolli L.A. (2007). Giant unilamellar vesicles electroformed from native membranes and organic lipid mixtures under physiological conditions. Biophys. J..

[28] Schindelin J., Arganda-Carreras I., Frise E., Kaynig V., Longair M., Pietzsch T., Preibisch S., Rueden C., Saalfeld S., Schmid B. (2012). Fiji: An open-source platform for biological-image analysis. Nat. Methods.

[29] McConnell H.M., Watts T.H., Weis R.M., Brian A.A. (1986). Supported planar membranes in studies of cell-cell recognition in the immune system. Biochim. Biophys. Acta Rev. Biomembr..

[30] Hope M.J., Bally M.B., Webb G., Cullis P.R. (1985). Production of large unilamellar vesicles by a rapid extrusion procedure. Characterization of size distribution, trapped volume and ability to maintain a membrane potential. Biochim. Biophys. Acta Biomembr..

[31] Spearman P., Horton R., Ratner L., Kuli-Zade I. (1997). Membrane binding of human immunodeficiency virus type 1 matrix protein in vivo supports a conformational myristyl switch mechanism. J. Virol..

[32] Wittig I., Braun H., Schägger H. (2006). Blue native PAGE. Nat. Protoc..

[33] Mukaka M.M. (2012). Statistics corner: A guide to appropriate use of correlation coefficient in medical research. Malawi Med. J..

[34] Bleicken S., Landeta O., Landajuela A., Basañez G., García-Sáez A.J. (2013). Proapoptotic Bax and Bak proteins form stable protein-permeable pores of tunable size. J. Biol. Chem..

[35] Apellániz B., Nieva J.L., Schwille P., García-Sáez A.J. (2010). All-or-none versus graded: Single-vesicle analysis reveals lipid composition effects on membrane permeabilization. Biophys. J..

[36] Schön P., García-Sáez A.J., Malovrh P., Bacia K., Anderluh G., Schwille P. (2008). Equinatoxin II permeabilizing activity depends on the presence of sphingomyelin and lipid phase coexistence. Biophys. J..

[37] Ruan Y., Rezelj S., Bedina Zavec A., Anderluh G., Scheuring S. (2016). Listeriolysin O Membrane Damaging Activity Involves Arc Formation and Lineaction–Implication for Listeria monocytogenes Escape from Phagocytic Vacuole. PLoS Pathog..

[38] Heyman N.S., Burt J.M. (2008). Hindered diffusion through an aqueous pore describes invariant dye selectivity of Cx43 junctions. Biophys. J..

[39] Masin J., Fiser R., Linhartova I., Osicka R., Bumba L., Hewlett E.L., Benz R., Sebo P. (2013). Differences in purinergic amplification of osmotic cell lysis by the pore-forming RTX toxins *Bordetella pertussis* CyaA and Actinobacillus pleuropneumoniae ApxIA: The role of pore size. Infect. Immun..

[40] Fuertes G., Giménez D., Esteban-Martín S., Sánchez-Muñoz O.L., Salgado J. (2011). A lipocentric view of peptide-induced pores. Eur. Biophys. J..

[41] Cannella S.E., Ntsogo Enguéné V.Y., Davi M., Malosse C., Sotomayor Pérez A.C., Chamot-Rooke J., Vachette P., Durand D., Ladant D., Chenal A. (2017). Stability, structural and functional properties of a monomeric, calcium-loaded adenylate cyclase toxin, CyaA, from *Bordetella pertussis*. Sci. Rep..

[42] Van Meer G., Voelker D.R., Feigenson G.W. (2008). Membrane lipids: Where they are and how they behave. Nat. Rev. Mol. Cell Biol..

[43] Lomize A.L., Pogozheva I.D., Mosberg H.I. (2011). Anisotropic solvent model of the lipid bilayer. 2. Energetics of insertion of small molecules, peptides, and proteins in membranes. J. Chem. Inf. Model.

[44] Hewlett E.L., Donato G.M., Gray M.C. (2006). Macrophage cytotoxicity produced by adenylate cyclase toxin from *Bordetella pertussis*: More than just making cyclic AMP!. Mol. Microbiol..

[45] Terrones O., Antonsson B., Yamaguchi H., Wang H., Liu J., Lee R.M., Herrmann A., Basañez G. (2004). Lipidic pore formation by the concerted action of proapoptotic BAX and tBID. J. Biol. Chem..

[46] Fišer R., Konopásek I. (2009). Different modes of membrane permeabilization by two RTX toxins: HlyA from *Escherichia coli* and CyaA from *Bordetella pertussis*. Biochim. Biophys. Acta Biomembr..

[47] Benz R., Hardie K.R., Hughes C. (1994). Pore formation in artificial membranes by the secreted hemolysins of Proteus vulgaris and Morganella morganii. Eur. J. Biochem..

[48] Tweten R.K. (2005). Cholesterol-dependent cytolysins, a family of versatile pore-forming toxins. Infect. Immun..

[49] Tweten R.K., Hotze E.M., Wade K.R. (2015). The Unique Molecular Choreography of Giant Pore Formation by the Cholesterol-Dependent Cytolysins of Gram-Positive Bacteria. Annu. Rev. Microbiol..

[50] Salvador-Gallego R., Mund M., Cosentino K., Schneider J., Unsay J., Schraermeyer U., Engelhardt J., Ries J., García-Sáez A.J. (2016). Bax assembly into rings and arcs in apoptotic mitochondria is linked to membrane pores. EMBO J..

[51] Cosentino K., Ros U., García-Sáez A.J. (2016). Assembling the puzzle: Oligomerization of α-pore forming proteins in membranes. Biochim. Biophys. Acta (BBA) Biomembr..

