# Supplementary material for: Membrane Permeabilization by Bordetella Adenylate Cyclase Toxin Involves Pores of Tunable Size
Source: Biomolecules. 2019 May 10;9(5):183. doi: 10.3390/biom9050183 (PMC6572617; doi:10.3390/biom9050183)
Supplement: Supplementary file 1 [file biomolecules-09-00183-s001.pdf]

SUPPLEMENTARY INFORMATION

Title: *Membrane permeabilization by the Bordetella Adenylate Cyclase Toxin involves growing-size pores of toroidal characteristics*

Authors: *David González-Bullón, Kepa B. Uribe, Eneko Largo, Garazi Genbelzu, Aritz B. García Arribas, César Martín and Helena Ostolaza*

Supplementary Figure S1. Permeabilization of GUVs for the 10kDa-Dextran-FITC determined at different incubation times and toxin concentrations. DOPC giant unilamellar liposomes (GUVs) at RT were exposed to control buffer (CTL) or to ACT toxin at three different concentrations (50, 200 and 500 nM) and for different times (30 to 120 min). The external medium contained the fluorescently labelled 10 kDa-Dextran-FITC in buffer with $CaCl_2$ 10 mM. Vesicle permeabilization (influx of the fluorescent solute) was visualized by confocal microscopy. The filling degree of the vesicles, represented in the figure as percentage of permeabilization, was calculated, for each incubation time and each toxin concentration, from the fluorescence values obtained from ≈200 individual vesicles, as detailed in the Experimental Procedures section.

Supplementary Figure S2. . Analysis by AFM of control POPC and ACT:POPC supported bilayers. AFM image of a supported lipid bilayer extended from a control sample of POPC liposomes, or from ACT:POPC proteoliposomes, and analyzed with AFM (room temperature). The topographical analysis of the bilayer is shown below each one of the images. On the right side of each panel a lateral view is shown.

Supplementary Figure S3. Correlation between the monomeric particle diameter and particle height for the different ACT assemblies visualized by atomic force microscopy. Graphical representations of the correlation between the monomeric particle diameter (in nm) and the monomeric particle height (in nm) for the different ACT assemblies (monomer, lines, arcs and full rings) visualized by atomic force microscopy.

Supplementary Figure S4. Comparative analysis by BN-PAGE of CR3 (+) and CR3(-) cells exposed to ACT. CR3(+) J774A.1 cells (1 x 10^6 cells/ml) or CR3(-) cells (1 x 10^6 cells/ml) were incubated with ACT (30 nM) at 37ºC and then the separated membranes were electrophoresed by BN-PAGE, blotted into a nitrocellulose membrane and stained with anti-ACT MAb 9D4. Several protein bands of apparent high molecular masses (≈800 and 1000 kDa) were resolved in both cell membranes, corresponding most likely to ACT oligomers of variable stoichiometry.

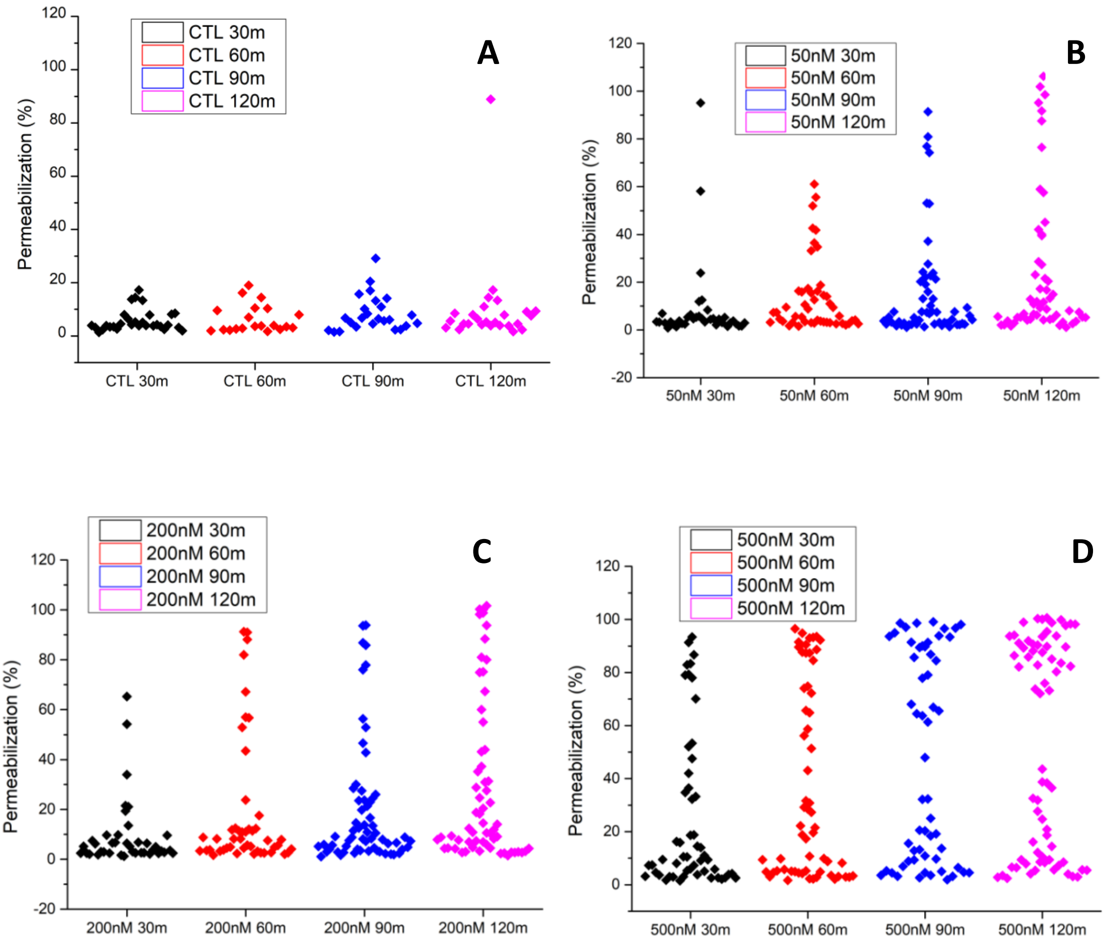

A
CTL 30m
CTL 60m
CTL 90m
CTL 120m
Permeabilization (%)
B
50nM 30m
50nM 60m
50nM 90m
50nM 120m
Permeabilization (%)
C
200nM 30m
200nM 60m
200nM 90m
200nM 120m
Permeabilization (%)
D
500nM 30m
500nM 60m
500nM 90m
500nM 120m
Permeabilization (%)

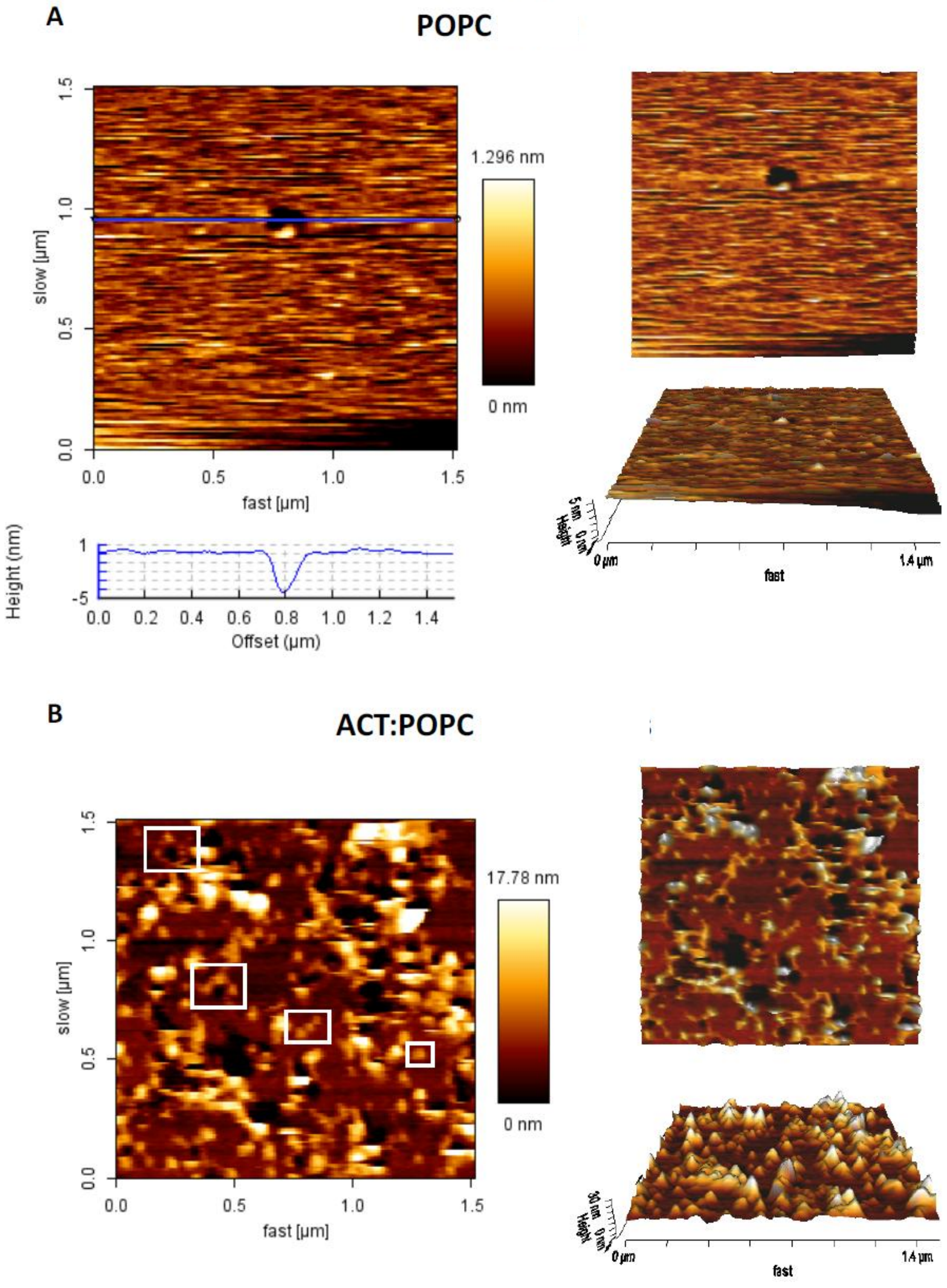

A
POPC
1.296 nm
0 nm
slow [µm]
fast [µm]
Height (nm)
Offset (µm)
fast
0 µm
1.4 µm
B
ACT:POPC
17.78 nm
0 nm
slow [µm]
fast [µm]
fast
0 µm
1.4 µm

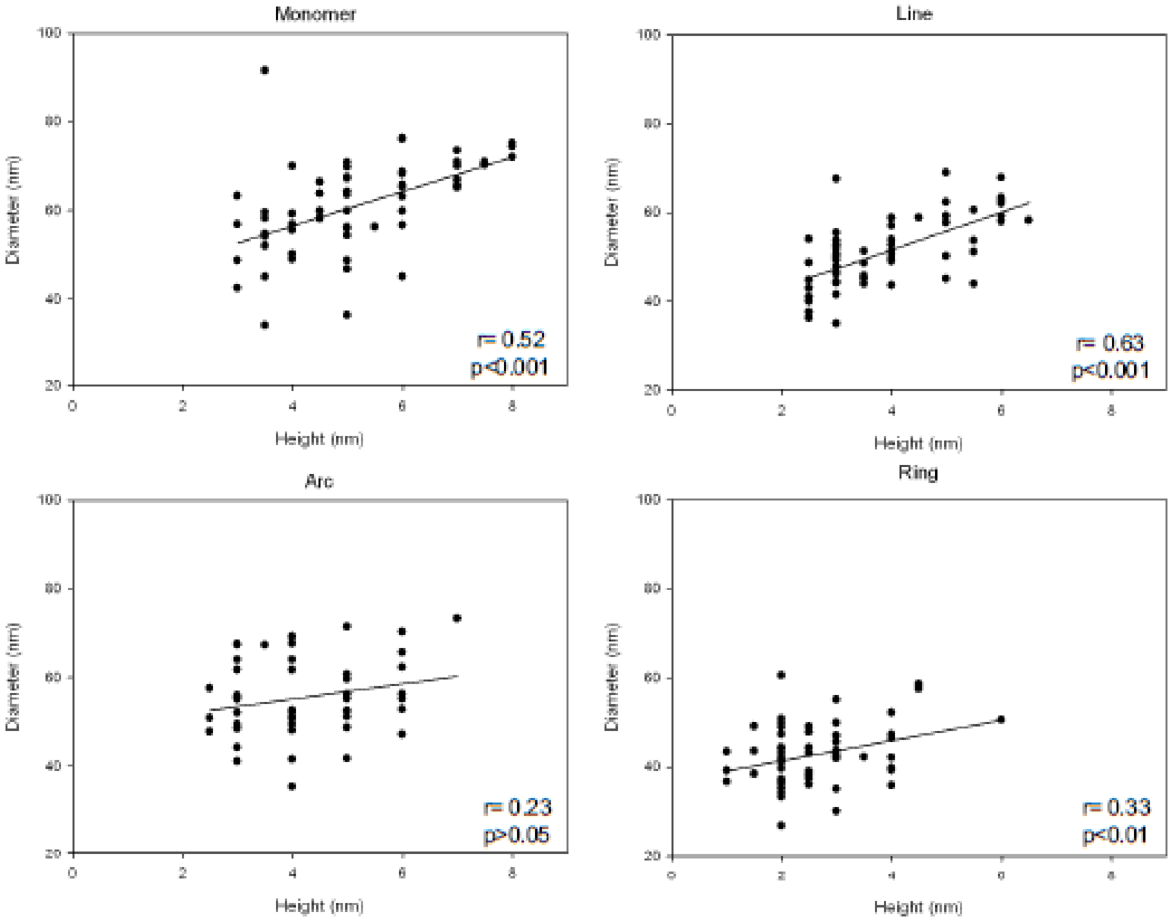
Monomer
Diameter (nm)
Height (nm)
r= 0.52
p<0.001
Line
Diameter (nm)
Height (nm)
r= 0.63
p<0.001
Arc
Diameter (nm)
Height (nm)
r= 0.23
p>0.05
Ring
Diameter (nm)
Height (nm)
r= 0.33
p<0.01

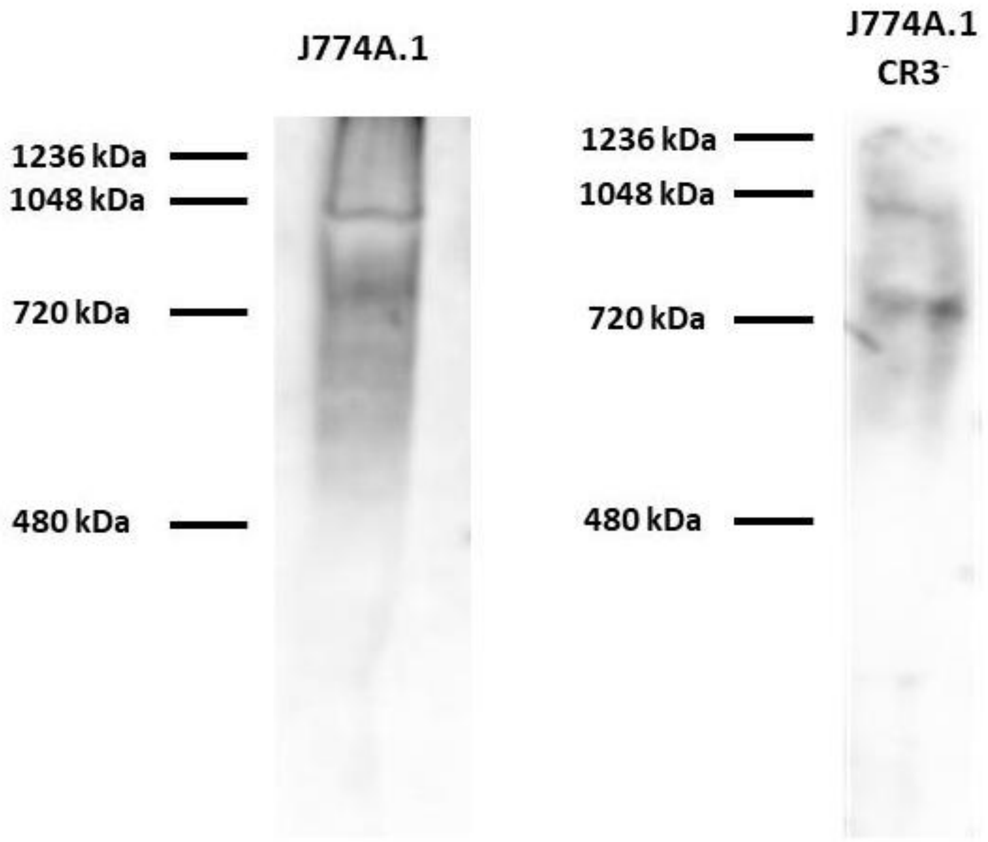
J774A.1
1236 kDa
1048 kDa
720 kDa
480 kDa
J774A.1
CR3-
1236 kDa
1048 kDa
720 kDa
480 kDa
